# The status of cervical cytology in Swaziland, Southern Africa: A descriptive study

**DOI:** 10.4103/1742-6413.54916

**Published:** 2009-08-06

**Authors:** Sylvain Okonda, Colleen Wright, Pam Michelow

**Affiliations:** Central Public Health Laboratory, Ministry of Health and Social Welfare, Swaziland; 1 Division of Anatomical Pathology, Department of Pathology, Faculty of Health Sciences, University of Stellenbosch and National Health Laboratory Service, Cape Town, South Africa; 2 Cytology Unit, Department of Anatomical Pathology, University of the Witwatersrand and National Health Laboratory Service, Johannesburg, South Africa

**Keywords:** Cervical screening, low resource communities, HPV

## Abstract

**Background:**

Cancer of the cervix is the most common cancer in women in Swaziland where most women never undergo cervical screening. The extremely high prevalence of HIV/AIDS in Swaziland complicates the management of preinvasive and invasive cervical cancer. The purpose of this study was to assess the current status of cervical cytology in Swaziland, its strengths and limitations.

**Methods:**

The study is a retrospective review of 12,188 conventional cervical smears received by the Central Public Health Laboratory in Swaziland from June 2004 to May 2006.

**Results:**

Review of results showed very high rates of cytologic abnormalities with 43.2% of smears screened reported as abnormal. The percentages of abnormalities were as follows: atypical squamous cells of undermined significance (ASC-US), 19.8%; atypical squamous cells, cannot exclude HSILs (ASC-H), 8.8%; low-grade squamous intraepithelial lesions (LSIL), 9.0%; high-grade squamous intraepithelial lesions (HSIL), 4.6%; squamous cell carcinomas, 0.5%; atypical endocervical cells, 0.6%; and atypical endometrial cells, 0.4%. Just over 5% of smears were inadequate. The highest rates of HSILs and invasive squamous carcinoma occurred in women aged 50–59 years.

**Conclusions:**

This study underscores the need to reduce the incidence of cervical cancer and its precursor lesions in Swaziland women. Based on studies of human papillomavirus (HPV) types in other Southern African countries, current HPV vaccines would reduce the incidence and mortality from cervical cancer in the future, but cervical screening would still be required, both for women already infected with the HPV and for HPV subtypes not covered by current vaccines. The most cost-effective combination of screening modalities such as visual inspection, HPV DNA testing, and cytology should be investigated. Cervical cancer reduction needs to be managed within the greater framework of the HIV/AIDS epidemic.

## INTRODUCTION

Cervical cancer is an important public health problem. Worldwide, as the second most common cancer in women, it comprises approximately 12% of all cancers but is the most common in developing countries.[1] The most recent compilation of global data indicates that an estimated 490,000 new cases of cervical cancer occur annually among women worldwide[2] and nearly 80% of these are in developing countries, where screening programs are not well established and are poorly organized. Worldwide, cervical cancer is the leading cause of cancer deaths among women and takes the lives of 270,000 women annually, with over 85% of these deaths occurring in developing countries.[3] Cervical screening programs, where effectively implemented, have reduced the incidence and mortality of cervical cancer dramatically, e.g., British Columbia, Aberdeen Scotland, Cali Columbia, Nordic European countries, and more recently Chile.[4]

The Kingdom of Swaziland is a small, independent, low-resource nation in Southern Africa. It covers an area of 17,364 km^2^ and is landlocked, with Mozambique to its east and South Africa to its north, west, and south.[5] The 2007 population and housing census estimated the population of Swaziland to be 1,018,449 with 537,021 females, including 126,616 aged between 30 and 59 years.[6] Currently, there are five gynecologists, one oncologist, three cytotechnicians, and one pathologist in Swaziland's government health institutions. In 1983, for the first time, cytopathology become available in Swaziland at the Central Public Health Laboratory. This remains the sole Cytopathology Laboratory in the country, receiving cervical smears from all health institutions (government, mission, and private hospitals as well as clinics and health centers). These cervical smears are in symptomatic women, i.e., presenting with vaginal discharge or lower abdominal pain, and only the Family Life Association of Swaziland offers cervical screening on a small scale, covering the two main towns of the country (Mbabane and Manzini). Many of these smears are, inappropriately, in women below 30 years of age.

Therefore, Swaziland falls into the category of countries without a well-established and organized cervical cancer screening program. In Swaziland, cancer of the cervix is the commonest cancer in women and its incidence is increasing. At the end of 1983 (ASIR 28.2),[7] it was 16.6 per 100,000 and 33.4 per 100,000 at the end of 1999 (ASIR 59.3),[8] recorded in a pathology-based cancer registry[9] and a population-based cancer registry.[7,8] This problem is complicated by the HIV/AIDS epidemic, as the prevalence of HIV/AIDS infection in Swazi adults was estimated to be 25.3, 38.2, and 42.4% in 1999, 2001, and 2003, respectively.[10] The management of HIV utilizes scarce public health resources, compromising other health interventions such as cervical screening. The incidence and pattern of cervical cancer in Swaziland is characterized by the presentation of precursor lesions at a young age (30–40 years).

The purpose of this study was to assess the current status of cytology in Swaziland and to determine its strengths and limitations. As far as can be determined, there are no other publications on cervical screening in Swaziland.

## MATERIALS AND METHODS

The study is based on a retrospective review of conventional cervical smears received, processed, and reported in the Department of Anatomical Pathology at the Central Public Health Laboratory from June 2004 to May 2006. As this laboratory is the only one in the country, these data are representative of the status of cervical smear pathology in the country. Written permission to undertake this study was obtained from all relevant health authorities, in addition to ethics permission being granted.

### Screening guidelines

To improve sensitivity of cervical screening by reducing the number of false negative smears, instructions and procedures as provided by several articles and books[11,12] were scrupulously adhered to. Requisition forms were sent to health facilities with instructions on how to prepare a client, when and how to collect a specimen, and how to submit a specimen for cytology.[11] Grossly visible lesions, including irregular, discolored, or friable areas were directly sampled and placed on a separate slide, especially if the lesion was distant from the transformation zone (TZ).

The microscopic examination of cervical smears was performed by registered cytotechnicians. All the findings were recorded and classified according to the 2001 Bethesda System.[13] All slides that were negative for intraepithelial lesion/malignancy were reported and signed out by cytotechnicians. Those with epithelial cell abnormalities were referred to the pathologist for final interpretation. Relevant patient information provided on the requisition was included in the demographic and clinical history sections of the final report. This assisted the clinician in correlating cytologic and clinical findings.

### Quality assurance

Quality assurance was performed by cytologic–histologic correlations, follow-up cytologic material and follow-up information on the patient. The full rescreening of a 10% random sample of slides originally reported as negative or a rapid review of 100% reportedly negative smears was attempted as far as possible, but was not always feasible given the severe staff shortages.

All premalignant and malignant cytology reports were correlated with subsequent histopathology reports. Cytohistologic correlations were a helpful educational tool in the diagnosis of both cytology and tissue specimens.[14] The correlation was documented in the Laboratory Quality Assurance Program. Where histologic material was not available, the Laboratory attempted to obtain follow-up cytologic material or information on patients. This was regularly achieved by sending a letter to the attending clinician requesting follow-up information. The report also included recommendations to the clinician whether to repeat the cytology after a prescribed time interval or after treatment, or for tissue studies to further evaluate epithelial cell abnormalities. Cytologic results were correlated with histologic findings in order to assess the accuracy of cytology in detecting cervical lesions, histology being considered the gold standard, although not the perfect one. The variation of accuracy tests was estimated according to the low-grade squamous intraepithelial lesion (LSIL) and high-grade squamous intraepithelial lesion (HSIL) thresholds and whether glandular cell lesions were included or not.

### Data analysis

Data were analyzed using CanReg4 package, a software program provided free of charge by the International Agency for Research on Cancer, based in Lyon, France. It is a variant of EPI.INFO, but specifically designed for cancer registries.[15]

## RESULTS

A total of 12,323 Pap smears were screened between 2004 and 2006. Fifty-five (0.43%) were broken beyond repair and 567 (4.48%) reported to be inadequate because they were obscured by purulent exudates (17%) or blood (21%), without transformation zone cells (42%) or with air-dried artifact (20%) [Table 1].

**Table 1:** Number of cervical smears per year

*Year*	*2004*	*2005*	*2006*	*Total (%)*
Number of cases	3,718	3,923	4,682	12,323 (100)
Broken slides	23	18	14	55 (0.45)
Inadequate smears	267	190	110	567 (4.60)
Adequate smears	3,428	3,715	4,558	11,701 (94.95)

The number of inadequate slides decreased with time (2.2, 1.5, and 0.9% in 2004, 2005, and 2006 respectively). This was the result of adopting the Bethesda System for reporting cervical cytology, as an adequacy statement was included which provided important feedback to clinicians regarding specimen collection and preparation techniques, and contributed to continuous quality improvement. The patient age varied between 18 and 88 years with a peak at 43 years. The age was unknown in 1.18% of cases.

The number of Pap smears reported to be adequate was 11,701, and the distribution by category diagnosis and age group is displayed in Table 2.

**Table 2:** Distribution of adequate epithelial cell abnormalities by age (in years) and category diagnosis (percentages in parentheses)

	*Unknown age*	*0–19*	*20–29*	*30–39*	*40–49*	*50–59*	*60–69*	*70–79*	*≥80*	*Total (%)*
NILM	60 (43.5)	378 (99.2)	503 (37.0)	2,261 (65.5)	1,218 (50.8)	879 (53.8)	588 (52.8)	423 (58.7)	278 (55.7)	6,588 (56.3)
ASC-US	32 (23.2)	3 (0.8)	594 (43.6)	531 (15.4)	364 (15.2)	262 (16.0)	222 (19.9)	161 (22.3)	149 (29.9)	2,318 (19.8)
ASC-H	22 (15.9)	0 (0.0)	78 (5.7)	257 (7.4)	386 (16.1)	131 (8.0)	81 (7.3)	53 (7.4)	18 (3.6)	1,026 (8.8)
LSIL	7 (5.1)	0 (0.0)	144 (10.6)	258 (7.5)	246 (10.3)	174 (10.6)	141 (12.7)	45 (6.2)	39 (7.8)	1,054 (9.0)
HSIL	6 (4.4)	0 (0.0)	23 (1.7)	99 (2.9)	132 (5.5)	161 (9.8)	64 (5.7)	29 (4.0)	13 (2.6)	527 (4.5)
SC Ca	2 (1.4)	0 (0.0)	0 (0.0)	7 (0.2)	10 (0.4)	16 (1.0)	9 (0.8)	7 (1.0)	2 (0.4)	53 (0.5)
AGCEndcx	5 (3.6)	0 (0.0)	14 (1.0)	21 (0.6)	24 (1.0)	2 (0.1)	1 (0.1)	0 (0.0)	0 (0.0)	67 (0.6)
AGCEndm	3 (2.2)	0 (0.0)	6 (0.4)	19 (0.6)	16 (0.7)	2 (0.1)	0 (0.0)	0 (0.0)	0 (0.0)	46 (0.4)
Endcxadenoca	1 (0.7)	0 (0.0)	0 (0.0)	0 (0.0)	0 (0.0)	4 (0.2)	5 (0.4)	3 (0.4)	0 (0.0)	13 (0.1)
EndmetadenoCa	0 (0.0)	0 (0.0)	0 (0.0)	0 (0.0)	2 (0.1)	4 (0.2)	3 (0.3)	0 (0.0)	0 (0.0)	9 (0.1)
Total	138 (100.0)	381 (100.0)	1,362 (100.0)	3,453 (100.0)	2,398 (100.0)	1,635 (100.0)	1,114 (100.0)	721 (100.0)	499 (100.0)	11,701 (100)

NILM = negative for intraepithelial lesion or malignancy; ASC-US = atypical squamous cell of undermined significance; ASC-H = atypical squamous cell, cannot exclude HSIL; LSIL = low-grade squamous intraepithelial lesion; HSIL = high-grade squamous intraepithelial lesion; SC Ca = squamous cell carcinoma; AGCEndcx = atypical glandular cells (endocervix); AGCEndmt = atypical glandular cells (endometrium); Endcxadenoca = endocervical adenocarcinoma; EndmetadenoCa = endometrial adenocarcinoma

The greatest numbers of atypical squamous cells of undermined significance (ASC-US) were seen in the age group 20–29 years while LSILs were mostly seen in the 20–29 and over–40 age group. This is an unusual finding in older women as LSIL rates seem to decrease in women over the age of 35 years. The greatest percentage of HSILs was seen in women over the age of 50 years.

From the 11,701 adequate Pap smears, 252 (2.2%) and 303 (2.6%) cases, respectively, at LSIL and HSIL thresholds were correlated with histologic findings in order to evaluate the accuracy of cytology in detecting cervical lesions; histology being considered the gold standard, although not the perfect diagnostic test. Using the LSIL threshold, 114 (45.2%) women were tested positive. The proportion of positive women decreased to 68 (22.4%) when the HSIL threshold was used. The distribution of histopathology findings by LSIL and HSIL thresholds are given in Tables 3 and 4 respectively.

**Table 3:** Histopathology findings at the LSIL threshold

*Cytology*	*Normal*	*Mild dysplasia*	*Moderate dysplasia*	*Severe dysplasia/carcinoma-in-situ*	*Invasive squamous Ca*	*Glandular cell abnormalities*	*Total*
Negative	106	8	12	6	5	1	138
Positive	52	14	5	3	7	33	114
Total	158	22	17	9	12	34	252

**Table 4:** Histopathology findings at the HSIL threshold

*Cytology*	*Normal*	*Mild dysplasia*	*Moderate dysplasia*	*Severe dysplasia/carcinoma-in-situ*	*Invasive squamous Ca*	*Glandular cell abnormalities*	*Total*
Negative	226	2	3	2	1	1	235
Positive	2	5	14	21	12	14	68
Total	228	7	17	23	13	15	303

At the LSIL threshold [Table 3], there were 114 cases of histologically proven LSILs or worse (true-positive cases). On further follow-up, cytology missed 23 cases, mainly glandular lesions (5 cases of endocervical carcinoma *in situ*, 4 cases of endocervical adenocarcinoma, 4 cases of endometrial adenomatous hyperplasia, 3 cases of endometrial adenocarcinoma, 4 cases of moderate cervical intraepithelial neoplasia, 2 cases of squamous carcinoma *in situ*, and 1 case of invasive squamous carcinoma). Additional training is required for more accurate cytologic diagnosis of glandular lesions. Fifteen smears initially positive were negated in the follow-up and confirmed as such in histology. They were five cases of reparative changes, three cases of ASC-US, four cases of immature squamous metaplasia, and three of infection (cytomegalovirus and herpes simplex virus).

The accuracy of detecting cervical lesions at the LSIL threshold (excluding glandular cell abnormalities) was 82.8% for sensitivity, 91.3% for specificity, 65.8% for the positive predictive value, and 96.3% for the negative predictive value. When glandular cell abnormalities were taken into consideration, the accuracy of detecting cervical lesions at the LSIL threshold was 64.5% for sensitivity, 83.1% for specificity, 55.5% for the positive predictive value, and 87.7% for the negative predictive value. Most discrepancies between cytology and histology follow-up were, on review, found to be due to sampling errors.

At the HSIL threshold [Table 4], there were 68 cases of histologically proven HSILs or worse (true-positive cases). On further follow-up, cytology missed 15 cases, mainly glandular lesions (5 cases of endocervical carcinoma *in situ*, 3 cases of endometrial carcinoma *in situ*, 2 cases of endometrial adenocarcinoma, 3 cases of severe cervical intraepithelial neoplasia, and 2 cases of microinvasive squamous cell carcinoma). Five smears initially positive were negated in the follow-up and confirmed as such in histology. They were two cases of reparative changes, one case of immature squamous metaplasia, one case of cytomegalovirus infection, and one case of fungal infection, probably coccidioidomycosis.

The accuracy of detecting cervical lesions at the HSIL threshold (excluding glandular cell lesions) was 80.7% for sensitivity, 93.8% for specificity, 58.3% for the positive predictive value, and 97.8% for the negative predictive value. When glandular cell abnormalities were taken into consideration, the accuracy of detecting cervical lesions at the HSIL threshold was 43.5% for sensitivity, 90.8% for specificity, 46.5% for the positive predictive value, and 89.8% for the negative predictive value.

The HIV serostatus of women undergoing cervical cytology is currently unknown. This will be the subject of a further study.

## DISCUSSION

The Cytopathology Service is equipped to screen 4,000 smears annually. It is located at the Central Public Health Laboratory with reliable transport of slides and test results to and from the Laboratory. Most of the cervical smears screened (>34%) are taken for diagnosis rather than for screening of cervical lesions. A well-established and well-organized cervical cancer prevention program does not exist and is urgently needed since 43.2% of smears screened are abnormal (19.8% ASC-US, 8.8% ASC-H, 9.0% LSILs, 4.6% HSILs, 0.5% squamous carcinoma, 0.6% atypical endocervical cells, and 0.4% atypical endometrial cells). Several reports from other Southern African nations also show high rates of cervical cytologic abnormalities. A study from Zimbabwe noted 59% abnormal smears with 12% LSILs and 3.6% HSILs.[16] A cross-sectional study of 22,160 cervical smears in a rural South African population showed a prevalence of LSILs of 8.3%, HSILs of 2.4%, and invasive carcinoma of 1.6%.[17] A study from Malawi showed squamous intraepithelial lesions in 15% of HIV-positive women and in 7% HIV-negative women.[18] A cervical smear study in HIV-positive women in Zambia detected extremely high rates of cervical abnormalities with 76% squamous intraepithelial lesions, 23.3% LSILs, 32.6% HSILs, and 20% suspicious for squamous carcinoma.[19] Correct management of these significant lesions is required to prevent invasive cancer from occurring in a substantial percentage of these women. Primary and secondary preventative programs for cervical cancer are justified and highly recommended.

Prevention of cervical cancer can be primary or secondary. Primary prevention modalities include changes in sexual behavior and human papillomavirus (HPV) vaccination. The former should be encouraged to prevent HIV, HPV, and other sexually transmitted diseases. The HPV serotypes in Swaziland have never been determined. There have been limited HPV studies in other Southern African nations that are probably relevant to Swaziland. The most common HPV type in Southern Africa appears to be HPV 16 that has a prevalence of between 17% and 82% depending on the type of specimen (cervicovaginal lavage, cervical smear, and biopsy) and underlying pathology (negative, preinvasive, or invasive disease). The prevalence of HPV 18 again varies in different Southern African studies from 1.6 to 21%. The prevalence (in parentheses) of other common HPV types in the region include HPV 52 (4–27%), HPV 53 (20%), HPV 58 (2–24%), HPV 51 (15%), HPV 45 (13%), HPV 31 (2–11%), HPV 33 (5–14%), and HPV 35 (2–10%).[20–26] In addition, a study by Ng'andwe in Zambia[21] showed a strong association between positive HIV status and the prevalence of high-risk HPV types especially HPV 18. A study by Baay *et al.* in Zimbabwe[23] found that certain HPV types (HPV types 11, 39, 43, 51, and 59) occurred more frequently in HIV-positive women suggesting that HIV coinfection may have an impact on HPV genotype distribution.[27]

Swaziland, as a Southern African nation, probably has similar rates of HPV infection and subtypes. Thus, it appears as if vaccination against HPV 16 and 18 in Swaziland would prevent many cases of cervical cancer in the future. However, given the high prevalence of HIV in Swaziland, the efficacy of HPV vaccination in HIV-positive women needs to be evaluated. It would be recommended to vaccinate girls before they are exposed to both HIV and HPV.

Secondary prevention of cervical cancer includes visual inspection of the cervix (VIA), cervicoscopy, HPV testing. and cytology.

The results of test accuracy in large cross-sectional, randomized controlled trials in developing countries indicate that the sensitivity of visual inspection of the cervix with acetic acid (VIA) to detect high-grade precancerous lesions ranges from 66 to 96% (median 84%); the specificity varied from 64 to 98% (median 82%); the positive predictive value ranged from 10 to 20% and the negative predictive value from 92 to 97%.[28,29] The major strengths of VIA include the following: its simplicity, can be taught to nurses, nurse-midwives, and other health workers; lower cost than other approaches in routine use; and real-time availability of results. The major limitations of VIA include a low specificity (generally less than 85%) and a low positive predictive value of the test.[27–30]

Cervicography or magnified visual inspection with acetic acid (VIAM) involves examination of magnified photographic documentation of the acetic acid-impregnated cervix. The results from cross-sectional studies established that VIAM does not improve the test performance of VIA over and above that of naked-eye visualization. Its sensitivity is lower than that of cytology and VIA to detect high-grade lesions, although the specificity is comparable to that of cytology.[30]

HPV testing has a high negative predictive value but low positive predictive value as the vast majority of women will clear an HPV infection spontaneously, especially women below the age of 30 years. Other disadvantages of HPV DNA testing are the cost; the dependence on reagents currently produced by very few commercial manufacturers; the requirement for a sophisticated and expensive laboratory equipment (molecular diagnostic laboratory); and trained personnel. Advantages of HPV DNA testing in developed countries include good inter- and intraobserver correlation; reliable quality assurance; and very high sensitivity. However, HPV DNA test as a primary screening method, at this time, is recommended for use only in pilot projects or other closely monitored settings. It can be used in conjunction with cytological or other screening tests, where sufficient resources exist.[31–34]

Cervical cytology is currently the only cervical screening method that is available in Swaziland. When detecting a cervical abnormality at the LSIL threshold (when glandular lesions were excluded), the sensitivity was 82.8%, specificity was 91.3%, positive predictive value was 65.8%, and negative predictive value was 96.3%. When detecting a cervical abnormality when HSIL was the threshold, the sensitivity was 80.7%, specificity was 93.8%, positive predictive value was 58.3%, and negative predictive value was 97.8%. This may improve with the implementation of more stringent internal and external quality assurance modalities.

Concerning the cost-effectiveness, Goldie *et al*.[35,36] studied the lifetime risk of cancer, years of life saved, lifetime costs, and cost-effectiveness ratios (cost per year of life saved) for each screening method in five developing countries having different cultural, geographical, and epidemiological backgrounds (India, Kenya, Peru, South Africa, and Thailand). They concluded that the most cost-effective strategies were those that required the fewest visits, resulting in improved follow-up testing and treatment.

A combination of screening methods may be appropriate to Swaziland, e.g., HPV DNA testing first and then cervical cytology of those patients found to be positive for high-risk HPV subtypes in urban women; while rural women may benefit from a combination of visual inspection and cervical cytology. Additional studies are required in this regard. Other areas for research include potential efficacy of HPV vaccination and cost-effectiveness of liquid-based cytology in Swaziland. Colposcopy and the whole arsenal of ablative (cryotherapy, cold coagulation, electrosurgical cauterization or vaporization with a laser beam, electrofulguration, laser ablation) and excisional (LEEP or LLETZ) surgery used for outpatients do not exist in Swaziland. Therefore, treatment services require significant strengthening, as only punch biopsy and hysterectomy are available.

There is an extremely high prevalence of HIV/AIDS in Swaziland. The prevalence of HIV/AIDS infection in adults was estimated to be 25.3, 38.2, and 42.4% in 1999, 2001, and 2003 respectively.[10] Women infected with the HIV virus have a higher prevalence of HPV infection, higher prevalence of persistent infection and infection with multiple high-risk HPV subtypes, higher prevalence of preinvasive cervical disease, increased risk of developing invasive cervical cancer, development of cervical cancer 10 or more years earlier than HIV-negative women, and higher recurrence of preinvasive and invasive cervical lesions after treatment. Thus, it is important that these women undergo some type of cervical cancer prevention program.[37–40] Unfortunately, the management of HIV utilizes scarce public health resources, leaving less for other health interventions such as cervical cancer preventive measures. Therefore, any health policy regarding the most efficient ways to reduce the incidence of and mortality from cervical cancer needs to be within the framework of the HIV/AIDS epidemic. It appears from studies in other Southern African countries that the current HPV vaccinations, given before girls are exposed to both HIV and HPV, would significantly reduce the incidence of and mortality from cervical cancer in Swaziland, although this reduction would only be seen in several decades. However, these vaccines at current pricing may be unaffordable to those who need it the most. Thus, safer sex practices and appropriate screening in conjunction with HPV vaccination offer the best means of reducing mortality from cervical cancer, both in the short and long term.

## COMPETING INTEREST STATEMENT BY ALL AUTHORS

There are no competing interests to declare by any of the authors.

## AUTHORSHIP STATEMENT BY ALL AUTHORS

Each author acknowledes that the final version was read and approved. According to International Committee of Medical Journal Editors (ICMJE http://www.icmje.org) “author” is generally considered to be someone who has made substantive intellectual contributions to a published study. Authorship credit should be based on (1) substantial contributions to conception and design, acquisition of data, or analysis and interpretation of data; (2) drafting the article or revising it critically for important intellectual content; and (3) final approval of the version to be published. Authors should meet conditions 1, 2, and 3. Other contributors, who do not meet these criteria for authorship, are listed in an acknowledgments section. All authors of this article declare that we qualify for authorship as defined by ICMJE http://www.icmje.org/#author. Each author has participated sufficiently in the work and takes public responsibility for appropriate portions of the content of this article.

## References

[1] Ferlay J, Bray F, Pisani P, Parkin DM (2002). International Agency for Research on Cancer (IARC). GLOBOCAN 2002: Cancer Incidence, Mortality and Prevalence Worldwide.

[2] Parkin D, Whelan S, Ferlay J (2002). Cancer Incidence in Five Continents.

[3] Steward BW, Kleihues P (2003). World Cancer Report.

[4] Sepulveda C, Prado R (2005). Effective cervical cytology screening in middle-income countries: The Chilean experience. Cancer Detect Prev.

[5] (2005). Geography Atlas for Swaziland.

[6] Swaziland population census 2007. Central Statistic Office.

[7] Peers F, Keen P, Parkin DM (1986). Swaziland Cancer Registry 1979-83. Cancer Occurrence in Developing Countries.

[8] Okonda SL, Parkin DM, Ferlay J, Hamdi-Cherif (2003). Population-based cancer registry in Swaziland, 1996-99. Cancer in Africa: Epidemiology and Prevention. IARC. Scientific Publication No 153.

[9] Okonda SL, Dlamini EM and Fraser MG (2003). Pathology-based cancer database in Swaziland, 1990-95. J Reg Mgmt.

[10] (2004). 2004 Report on the global AIDS epidemic: 4^th^ global report. UNAIDS.

[11] American Society of Cytopathology (2000). Cervical Cytology Practice Guidelines. Diagn Cytopathol.

[12] Koss L (1989). The Papanicolaou test for cervical cancer detection: a triumph and tragedy. JAMA.

[13] Solomon D, Davey D, Kurman R, Moriarty A, O'Connor D, Prey M (2002). American Medical Association. The 2001 Bethesda System: terminology for reporting results of cervical cytology. JAMA.

[14] Sodhani P, Singh V, Das DK, Bhambhani S (1997). Cytohistologal correlation as a measure of quality assurance of a cytology laboratory. Cytopathology.

[15] Available from: http://www.iarc.com.fr/canreg4.htm. [last accessed on 2008 June 19].

[16] Thistle PJ, Chirenje ZM (1997). Cervical cancer screening in a rural population of Zimbabwe. Centr Afr J Med.

[17] van Bogaert LJ, Knapp DC (2001). Opportunistic testing of medically underserved women for cervical cancer in South Africa. Acta Cytol.

[18] Motti PG, Dallabetta GA, Daniel RW, Canner JK, Chiphangwi JD, Liomba GN (1996). Cervical abnormalities, human papillomavirus and human immunodeficiency virus infections in women in Malawi. J Infect Dis.

[19] Parham GP, Sahasrabuddhe VV, Mwanahamuntu MH, Shepherd BE, Hicks ML, Stringer EM (2006). Prevalence and predictors of squamous intraepithelial lesions of the cervix in HIV infected women in Lusaka, Zambia. Gynecol Oncol.

[20] Castellsagué X, Klaustermeier J, Carrilho C, Albero G, Sacarlal J, Quint W (2008). Vaccine-related HPV genotypes in women with and without cervical cancer in Mozambique: Burden and potential for prevention. Int J Cancer.

[21] Ng'andwe C, Lowe JJ, Richards PJ, Hause L, Wood C, Angeletti PC (2007). The distribution of sexually-transmitted Human Papillomaviruses in HIV positive and negative patients in Zambia, Africa. BMC Infect Dis.

[22] Sahasrabuddhe VV, Mwanahamuntu MH, Vermund SH, Huh WK, Lyon MD, Stringer JS (2007). Prevalence and distribution of HPV genotypes among HIV-infected women in Zambia. Br J Cancer.

[23] Baay MF, Kjetland EF, Ndhlovu PD, Deschoolmeester V, Mduluza T, Gomo E (2004). Human papillomavirus in a rural community in Zimbabwe: the impact of HIV co-infection on HPV genotype distribution. J Med Virol.

[24] Mayaud P, Gill DK, Weiss HA, Uledi E, Kopwe L, Todd J (2001). The interrelation of HIV, cervical human papillomavirus and neoplasia among antenatal clinic attenders in Tanzania. Sex Transm Infect.

[25] Kay P, Soeters R, Nevin J, Denny L, Dehaeck CM, Williamson AL (2003). High prevalence of HPV 16 in South African women with cancer of the cervix and cervical intraepithelial neoplasia. J Med Virol.

[26] Marais DJ, Sampson CC, Urban MI, Sitas F, Wiliamson AL (2007). The seroprevalence of IgG antibodies to human papillomavirus (HPV) types HPV-16, HPV-18 and HPV-11 capsid-antigens in mothers and their children. J Med Virol.

[27] (2002). Cervical cancer screening in developing countries: report of a WHO consultation. World Health Organization 2002.

[28] Sankaranarayanan R, Gaffikin L, Jacob M, Sellors J, Robles S (2005). A critical assessment of screening methods for cervical neoplasia. Int J Gynecol Obstet.

[29] Shastri S, Dinshaw K, Amin G, Goswami S, Patil S, Chinoy R (2005). Concurrent evaluation of visual, cytology and HPV testing screening method in the early detection of cervical neoplasia in Mumbai, India. Bull World Health Organ.

[30] Sankaranarayanan R, Basu P, Wesley RS, Mahe C, Keita N, Mbalawa CC (2004). Accuracy of visual screening for cervical neoplasia: Results from an IARC multicentre study in India and Africa. Int J Cancer.

[31] Miller AB, Nazeer S, Fonn S, Brandup-Lukanow A, Rehman R, Cronje H (2000). Report on consensus conference on cervical cancer screening and management. Int J Cancer.

[32] Wright TC, Schiffman M, Solomon D, Cox JT, Garcia F, Goldie S (2004). Interim guidance for the use of human papillomavirus DNA testing as an adjunct to cervical cytology for screening. Obstet Gynecol.

[33] Howard M, Sellors J, Kaczorowski J (2002). Optimizing the hybrid capture II human papillomavirus test to detect cervical intraepithelial neoplasia. Obstetric Gynecol.

[34] Sellors J (2005). HPV in screening and triage: towards an affordable test. HPV Today.

[35] Goldie SJ, Gaffikin L, Goldhaber-Fiebert JD, Gordillo-Tobar A, Levin C, Mahé C (2005). Cost-effectiveness of cervical cancer screening in five developing countries. N Engl J Med.

[36] Goldie SJ, Kuhn L, Denny L, Pollack A, Wright TC (2001). Policy analysis of cervical cancer screening strategies in low-resource settings: clinical benefits and cost-effectiveness. JAMA.

[37] Palefsky JM, Holly EA (2003). Immunosuppression and co-infection with HIV. J Natl Cancer Inst Monogr.

[38] Maiman M, Fruchter RG, Guy L, Cuthill S, Levine P, Serur E (1993). Human immunodeficiency virus infection and invasive cervical cancer. Cancer.

[39] Maiman M, Frutcher RG, Clark M (1993). Cervical cancer as an AIDS-defining illness. Obstet Gynecol.

[40] Clark B, Chetty R (2002). Postmodern cancer: the role of human immunodeficiency virus in uterine cervical cancer. Mol Biol.

